# Simulation and Analysis of the Transient Absorption Spectrum of 4-(*N*,*N*-Dimethylamino)benzonitrile (DMABN) in Acetonitrile

**DOI:** 10.1021/acs.jpca.1c06166

**Published:** 2021-09-22

**Authors:** Michał Andrzej Kochman, Bo Durbeej, Adam Kubas

**Affiliations:** † Institute of Physical Chemistry, Polish Academy of Sciences, Ul. Marcina Kasprzaka 44/52, 01-224 Warszawa, Poland; ‡ Division of Theoretical Chemistry, Department of Physics, Chemistry and Biology (IFM), Linköping University, 581 83 Linköping, Sweden

## Abstract

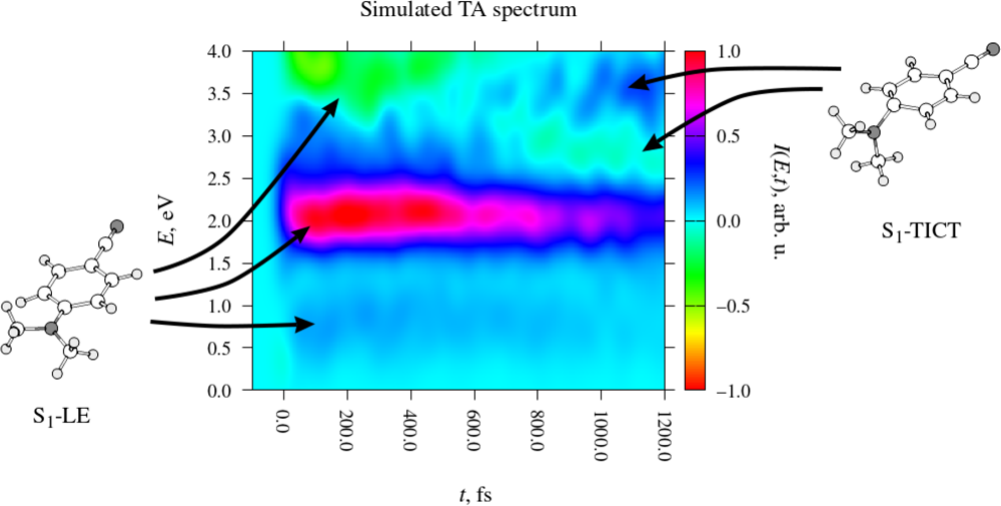

4-(*N*,*N*-Dimethylamino)benzonitrile (DMABN) is a well-known model compound for dual fluorescence—in sufficiently polar solvents, it exhibits two distinct fluorescence emission bands. The interpretation of its transient absorption (TA) spectrum in the visible range is the subject of a long-standing controversy. In the present study, we resolve this issue by calculating the TA spectrum on the basis of nonadiabatic molecular dynamics simulations. An unambiguous assignment of spectral signals to specific excited-state structures is achieved by breaking down the calculated spectrum into contributions from twisted and nontwisted molecular geometries. In particular, the much-discussed excited-state absorption band near 1.7 eV (ca. 700 nm) is attributed to the near-planar locally excited (LE) minimum on the S_1_ state. On the technical side, our study demonstrates that the second-order approximate coupled cluster singles and doubles (CC2) method can be used successfully to calculate the TA spectra of moderately large organic molecules, provided that the system in question does not approach a crossing between the lowest excited state and the singlet ground state within the time frame of the simulation.

## 1 Background

**Figure 1:**
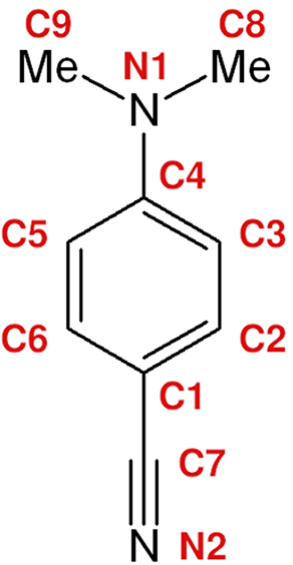
Molecular structure of 4-(*N*,*N*-dimethylamino)benzonitrile (DMABN). Atom numbering is given in red.

The simple molecular structure of 4-(*N*,*N*-dimethylamino)benzonitrile (DMABN, see Figure 1) belies a complex photophysics which has long held the attention of spectroscopists and theoreticians alike. This compound first came into the spotlight in 1959, when Lippert and co-workers^1,2^ discovered that it exhibits dual fluorescence, a rare phenomenon where a given fluorophore shows two distinct emission bands. In the case of DMABN, the first fluorescence band, known as the “normal” band, is present from shortly after the initial photoexcitation, and the position of its maximum is fairly insensitive to solvent polarity. In sufficiently polar solvents, the intensity of the normal band decreases on a time scale of a few picoseconds, while simultaneously a second fluorescence band appears. This latter band is termed the “anomalous” band. It is strongly red-shifted with respect to the normal band, and the position of its maximum is more sensitive to solvent polarity. The emission profile eventually stabilizes with both bands being present. The intensity ratio of the anomalous and normal bands increases with increasing solvent polarity.

**Figure 2:**
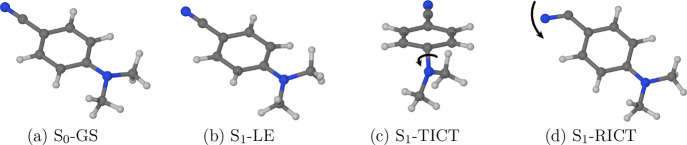
Visual catalogue of the ground- and excited-state structures of DMABN. The prefix S_0_- or S_1_- identifies the adiabatic state on which a given structure is located. The characteristic geometric features of the S_1_-TICT and the S_1_-RICT structures—the twisting of the dimethylamino group and the bending of the nitrile group, respectively—are indicated with arrows.

The discovery of the dual fluorescence process of DMABN attracted much attention, and this and related donor–acceptor compounds soon became the focus of intensive research. For a detailed historical perspective, we refer the reader to refs (3−6) and to the Background section of ref (7). Presently, we will restrict ourselves to a very brief overview of the current state of the understanding of the mechanism underlying the dual fluorescence of DMABN. Despite some past controversies, the following model now seems generally accepted: the dual fluorescence involves emission from two distinct excited-state species. Both of these exist as minima on the potential energy surface (PES) of the S_1_ adiabatic state, but they differ in terms of geometry and electronic structure. One is the locally excited structure (denoted S_1_-LE), which is depicted in Figure 2b. Its geometry is planar or near-planar, and its electric dipole moment is only slightly larger in magnitude than that of the ground-state structure (S_0_-GS). It gives rise to the normal emission band. The other is the twisted intramolecular charge transfer structure (S_1_-TICT, Figure 2c), so called because it is characterized by a rotation of the dimethylamino group with respect to the six-membered ring. The S_1_-TICT structure features a shift of electron density from the dimethylamino group nitrogen onto the benzonitrile moiety, leading to a large electric dipole moment, and it is responsible for the anomalous emission band.

The singlet-state lifetime of DMABN is long enough that the S_1_-LE and S_1_-TICT structures reach a quasi-equilibrium. In a nonpolar solvent, the S_1_-LE structure is the lower in energy and holds the vast majority of the excited-state population. Hence, only the normal emission band is observed. Polar solvation stabilizes the S_1_-TICT structure relative to the S_1_-LE structure, owing to which the anomalous band gains in intensity in polar solvents.

Although there is consensus that the anomalous band originates from the S_1_-TICT structure, the exact sequence of events leading to the formation of that species remains under debate. Much of the controversy can be traced back to the interpretation of the transient absorption (TA) spectrum of DMABN in the visible range. The spectrum itself was recorded independently by several groups,^8−15^ and its structure is not under debate; it is only the assignment of excited-state absorption (ESA) bands to specific molecular structures that is controversial. In nonpolar solvents such as *n*-hexane, the spectrum shows a strong ESA band with a maximum at around 1.65 eV (or 750 nm) and a weaker ESA band with a maximum at around 2.70 eV (460 nm). In acetonitrile, a polar organic solvent, both these bands are present at short time delays after photoexcitation, albeit the former is slightly blue-shifted, such that its maximum occurs at around 1.75 eV (710 nm). Both decay with a time constant of roughly 4 ps, while simultaneously new ESA bands appear with maxima at around 2.95 eV (420 nm, a very weak band) and at around 3.80 eV (325 nm, a strong band).

The ESA band located near 1.7 eV has especially high diagnostic value, as it is strong, sharp, and located in an uncrowded region of the TA spectrum. However, its assignment to a specific excited-state structure remains unresolved. There are two conflicting interpretations. The first, and chronologically the earlier, was put forward by Zgierski and Lim,^16,17^ who calculated the ESA transitions of DMABN using time-dependent density functional theory (TDDFT). These authors ascribed the ESA band at around 1.7 eV to the rehybridized intramolecular charge transfer structure (S_1_-RICT), another charge transfer structure on the S_1_ state, whose existence had previously been predicted by the theoretical studies of Sobolewski and Domcke.^18,19^ As illustrated in Figure 2d, the S_1_-RICT structure is characterized by a bent nitrile group and an in-plane orientation of the dimethylamino group. This species is also sometimes referred to as the πσ* structure. In the model formulated by Zgierski and Lim, the S_1_-RICT structure is formed shortly after photoexcitation.^16,17^ In nonpolar solvents, it persists along with the S_1_-LE structure for an extended period of time (at least 100 ps), whereas in polar solvents, it converts into the S_1_-TICT structure.^16,17^ This model was further developed in a number of subsequent spectroscopic and theoretical studies.^20−23^

The interpretation that assigns the ESA band near 1.7 eV to the S_1_-RICT structure was subsequently disputed by Zachariasse and co-workers.^13,14^ These authors studied the relaxation dynamics of photoexcited DMABN with the use of TA and time-resolved fluorescence spectroscopy. In ref (13) the TA spectrum was decomposed into bleach, stimulated emission (SE), and ESA contributions of the S_1_-LE and the intramolecular charge transfer (ICT) structures. (References (13 and 14) took a phenomenological approach, and did not make any statement regarding the geometry of the ICT structure.) In particular, the ESA band at around 1.7 eV was assigned to the S_1_-LE structure. The rapid decrease of the intensity of that band in a polar solvent was attributed to the conversion of the S_1_-LE structure into the ICT structure. Later on, Zachariasse et al.^14^ provided another argument against the assignment of the ESA band near 1.7 eV to the S_1_-RICT structure. Namely, it was demonstrated that similar ESA bands also appear in the TA spectra of several DMABN analogues in which the nitrile group is replaced by other electron-withdrawing groups that are unable to adopt RICT-type structures.^14^ To this we may add that Kochman and Durbeej^7^ have recently reported nonadiabatic molecular dynamics simulations of the relaxation process of DMABN both in the gas phase (a nonpolar environment) and in a water nanodroplet (a strongly polar environment). These simulations predicted that DMABN does not adopt the S_1_-RICT structure, whether in a polar or a nonpolar environment.^7^ This would rule out the possibility that the 1.7 eV band originates from the S_1_-RICT structure.

The problem of the interpretation of the TA spectrum was taken up in a theoretical study by Galván and co-workers.^24^ In an earlier work,^25^ these authors had optimized the ground- and excited-state geometries of DMABN in the gas phase and in two solvents: tetrahydrofuran, a moderately polar organic solvent, and water, a strongly polar solvent. In these studies, the electronic structure of DMABN was described using the complete active space self-consistent field^26^ (CASSCF) method, with single-point energies being calculated at the complete active space second-order perturbation^27^ (CASPT2) level. The solvent, in turn, was treated via the average solvent electrostatic potential from the molecular dynamics^28,29^ (ASEP/MD) model. In ref (24) the resulting gas-phase and solution-phase equilibrium geometries of the S_1_-LE structure were used as the basis for the calculation of the ESA transitions. The calculated vertical excitation energies matched up very well with the positions of the ESA band maxima.^24^ The ESA band near 1.7 eV was attributed to the S_1_ → S_3_ transition, and the S_3_ state was characterized as an intramolecular charge transfer state in which electron density is transferred from the dimethylamino group nitrogen (N1) onto the benzonitrile moiety. However, as the authors were careful to point out, the calculations did not reproduce the solvent shift of the ESA band near 1.7 eV.^24^ Experimentally, this band exhibits a slight blue shift on going from *n*-hexane (a nonpolar solvent) to acetonitrile (a polar solvent).^13^ In contrast, the calculations predicted a slight red shift of the first ESA band on going from the gas phase to water.^24^ This discrepancy was tentatively attributed to the approximations of the ASEP/MD solvent model.^24^

The present study aims to clarify the situation with regard to the TA spectrum of DMABN and especially the assignment of the much-discussed ESA band near 1.7 eV. To this end, we simulate the TA spectrum from first-principles and correlate the various spectral signals with specific excited-state structures. A particular strength of the simulation-based approach to interpreting the TA spectrum is that the simulated spectrum can be decomposed into contributions from different molecular geometries, which is not possible using the experimentally observed spectrum alone. In the case of DMABN, the obvious choice is to break down the spectrum into contributions from twisted and nontwisted molecular geometries. As we shall see, this technique enables a clear and unambiguous assignment of spectral signals.

On the methodological side, the simulation and theoretical analysis of TA spectra in the ultraviolet–visible range is a field of active development.^30−46^ The present work advances the state of the art in this area by introducing a practical algorithm for the simulation of the complete TA spectrum on the basis of nonadiabatic molecular dynamics (NAMD) simulations at the second-order algebraic diagrammatic construction^47,48^ (ADC(2)) level of theory.

The rest of this paper is organized as follows. First, we describe the simulation setup, with special regard to the technical details of the calculation of the TA spectrum. Having done so, we assess the accuracy of our methodology by reviewing the results of some high-level benchmark calculations. We then examine the excited-state relaxation process of DMABN in an acetonitrile nanodroplet, which serves as our model of a dilute acetonitrile solution. Finally, we move on to discuss the main results of this study: the simulated TA spectrum and the band assignment.

## 2 Computational Methods

### 2.1 Overview

Our aim in the present study was to simulate the TA spectrum of DMABN in polar solution at a high level of accuracy, so as to enable a direct comparison to the available spectroscopic data. The basis for the calculation of the spectrum was provided by a set of NAMD simulations of the relaxation process of photoexcited DMABN in acetonitrile solution. At this stage, we drew heavily on the simulation methodology developed previously in ref (7). The spectrum itself was calculated later, at the postprocessing stage.

For technical reasons, the solution phase was represented by placing a single molecule of DMABN at the center of an acetonitrile nanodroplet containing 500 solvent molecules. Acetonitrile was chosen as the solvent because it has been used in many experimental studies of the photophysics of DMABN. It is a polar aprotic solvent. The PESs for this system were constructed with the use of the hybrid quantum mechanics/molecular mechanics (QM/MM) method, and its time evolution was propagated as an ensemble of 45 NAMD trajectories. These simulations were performed within our existing “wrapper” program,^7^ which contains an interface to the electronic structure program Turbomole.^49^ At each time step of a simulated trajectory, the wrapper program generates a Turbomole input file, runs Turbomole, and then parses the output and extracts the relevant quantities: the state energies and gradients. Nonadiabatic coupling elements are calculated with the use of the wave function overlap program developed by Plasser and co-workers.^50,51^ A subroutine of the wrapper program also calculates the MM terms of the QM/MM energies and gradients. These quantities are then used to propagate the nuclear and electronic equations of motion.

### 2.2 Electronic Structure Calculations

In the course of the NAMD simulations, the ground electronic state of the DMABN molecule was described with the Møller–Plesset perturbation theory method of second order (MP2), while its excited states were calculated with the use of the ADC(2) method. The spin-opposite scaling^52,53^ (SOS) procedure was imposed in both the MP2 and the ADC(2) calculations. Using the SOS variant of ADC(2) is justified by the fact that it predicts the correct energy ordering of the S_1_-LE and S_1_-TICT structures of DMABN in the gas phase, whereas the standard variant (i.e., without a rescaling of the same- and opposite-spin contributions to the correlation energy) incorrectly places the S_1_-TICT structure below the S_1_-LE structure.^7^ In order to avoid confusion with the standard variants of the MP2 and ADC(2) methods, these calculations are here referred to by the acronyms SOS-MP2 and SOS-ADC(2). The scaling factors were set to the values proposed in ref (52) for use with the MP2 method: *c*_OS_ = 1.3 for the opposite-spin contributions and *c*_SS_ = 0 for the same-spin contributions.

The SOS-MP2 and SOS-ADC(2) calculations were performed with the program Turbomole, version 6.3.1.,^49^ taking advantage of the frozen core and resolution of the identity^54−57^ approximations. A restricted Hartree–Fock (RHF) reference determinant was used. The cc-pVDZ basis set^58^ was employed with the default auxiliary basis set.^59^

The simulation of the TA spectrum requires the calculation of transition dipole moments (TDMs) between excited states. In Turbomole, such a calculation is only implemented for the coupled cluster singles (CCS) and the second-order approximate coupled cluster singles and doubles^60^ (CC2) methods. Because the CC2 method is the higher of the two in the hierarchy of coupled cluster approximations and is closely related to the ADC(2) method,^48^ we decided to use that method for the calculation of the TA spectrum. The details of how that was accomplished are given in Section 2.5. The simulation parameters (choice of basis set, reference determinant, etc.) in the CC2 calculations were the same as in the SOS-ADC(2) calculations. As with the ADC(2) method, in all CC2 calculations we imposed the SOS procedure, and we denote this with the acronym SOS-CC2. The scaling parameters were the same as in the SOS-MP2 and the SOS-ADC(2) calculations.

Further on the subject of the choice of the electronic structure method for the calculation of the TA spectrum, we note that CC2 is a relatively low-level method. One particular limitation of CC2, which may come into effect in this context, is that it is not capable of providing a correct description of excited states with a significant contribution from doubly excited configurations. Transitions into such states may potentially appear in the TA spectrum, especially at high energies. In order to determine whether CC2 provides an accurate description of the photoabsorption spectra of the excited-state structures of DMABN, its performance was benchmarked against extended multistate complete active space second-order perturbation theory^61^ (XMS-CASPT2). This latter method is the highest-level electronic structure method that we are able to apply to the relatively large DMABN molecule, and it is expected to provide an accurate description of doubly excited states.

The XMS-CASPT2 calculations were performed with the program BAGEL,^62,63^ version 1.1.2. The active space of the reference CASSCF calculation consisted of six π- and π*-type orbitals of the six-membered ring, four π- and π*-type orbitals of the nitrile group, and the lone pair orbital of the dimethylamino group, for a total of 11 active space orbitals. The choice of active space orbitals is shown in Figure S1 in the Supporting Information. A state-averaging scheme was imposed in the reference CASSCF calculations with the inclusion of the lowest five singlet states (which is to say, S_0_–S_4_). A vertical shift of 0.5 *E*_h_ (hartree) was imposed at all times.^64^ Moreover, the so-called single-state single-reference (SS-SR) contraction scheme was used. The cc-pVDZ basis set was employed in combination with the default density fitting basis set from the BAGEL library.

The XMS-CASPT2 method can also be used to evaluate the performance of the SOS-ADC(2) level of theory for the relative energies of the excited-state structures of DMABN. Accordingly, we have performed additional benchmark calculations comparing the relative energies obtained with XMS-CASPT2, SOS-ADC(2), and some other electronic structure methods. The detailed description of these calculations is given in Section S2 of the Supporting Information.

Owing to its high intensity, the S_1_ → S_3_ transition of the S_1_-LE structure is especially important for the TA spectrum. Therefore, this transition was characterized by plotting the electron density difference map (EDDM), which is defined as the difference of the electron density of the final state and that of the initial state at the same nuclear geometry. Thus, the EDDM shows the redistribution of electron density upon vertical S_1_ → S_3_ excitation. In the present case, we calculated the EDDM both with XMS-CASPT2 and with SOS-CC2 relaxed densities.

### 2.3 QM/MM Calculations

**Figure 3:**
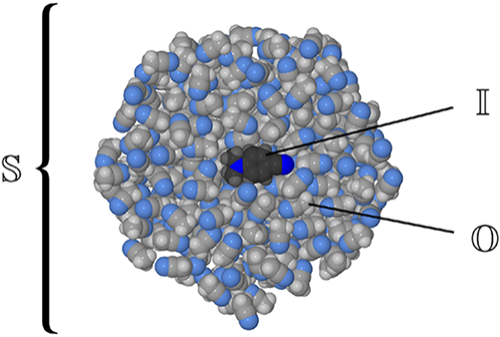
Schematic illustration of the partitioning of the system (denoted ) into the inner () and outer () subsystems. The figure shows a cross section of the 500-molecule acetonitrile droplet enclosing the DMABN molecule.

In simulations of condensed-phase systems, the standard approach is to model the bulk phase by imposing periodic boundary conditions (PBCs) on a finite system. However, PBCs are not implemented in the program Turbomole, and for this reason, in our simulations the solution phase was represented by immersing a single molecule of DMABN in a 500-molecule acetonitrile nanodroplet. The PESs for this system were constructed with the use of the additive variant of the QM/MM method.^65−67^ In this scheme, the system (denoted ) is partitioned into two subsystems which are treated at different levels of approximation. The electronic structure of the inner subsystem () is explicitly included in the calculation. The outer subsystem (), in turn, is described with the use of a molecular mechanics (MM) force field. As shown in Figure 3, in the present case the inner subsystem consisted of the DMABN molecule, and the acetonitrile molecules collectively comprised the outer subsystem.

In the course of the NAMD simulations, the electronic structure of the DMABN molecule was described with the SOS-ADC(2) method, and the acetonitrile molecules were treated with the all-atom optimized potentials for liquid simulations^68,69^ (OPLS-AA) force field. The van der Waals interactions between the DMABN molecule and the solvent molecules were described via the 12−6 Lennard-Jones potential. The interaction parameters for DMABN were also sourced from the OPLS-AA force field. For details on the choice of parameters, see the Supporting Information of ref (7). Moreover, the electrostatic interactions between the DMABN molecule and the solvent molecules were accounted for by including the point charges of the solvent molecules in the Hamiltonian of the inner subsystem.

### 2.4 NAMD Simulations

The NAMD simulations were carried out with the fewest switches surface-hopping (FSSH) algorithm,^70−73^ adapted for use in combination with the hybrid QM/MM method. In this approach, the nuclear wavepacket of the system is represented by an ensemble, or “swarm,” of mutually independent semiclassical trajectories. In each simulated trajectory, the nuclei are described by means of classical mechanics and evolve on the PES of a single adiabatic state: the so-called current state for that trajectory. Nonadiabatic effects are accounted for by allowing a trajectory to undergo a switch (or “hop”) between the current state and another adiabatic state, which then becomes the new current state for the given trajectory. In the present case, the S_1_ and S_2_ states were included in the FSSH algorithm.

**Figure 4:**
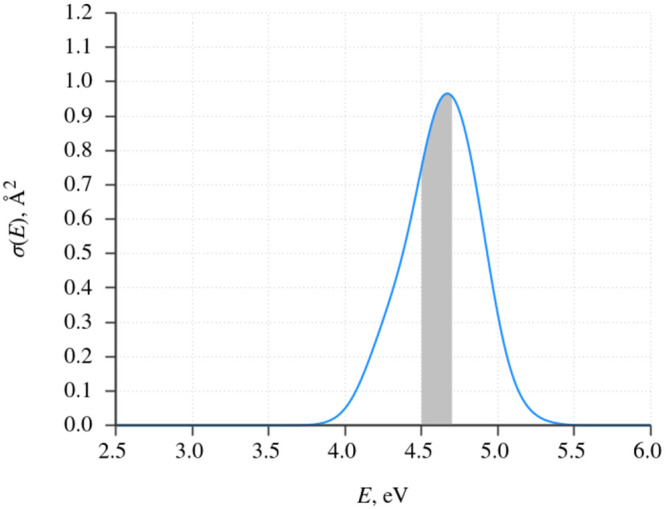
Simulated photoabsorption cross section of DMABN in the 500-molecule acetonitrile nanodroplet, calculated with the use of the nuclear ensemble method of Crespo-Otero and Barbatti.^74^ The shaded area represents the energy interval of 4.6 ± 0.1 eV, from which the initial conditions were sampled.

The initial conditions for the NAMD simulations were generated in such a way as to model photoexcitation by a laser pulse, analogously as in ref (7). The energy range of the photoexcitation pulse was set to 4.6 ± 0.1 eV, which covers the maximum of the simulated photoabsorption spectrum of DMABN in the acetonitrile droplet (see Figure 4). The simulated trajectories were propagated for a time period of 1.2 ps. The time evolution of the system was propagated with a multitime-step procedure. The dynamics of the nuclei was propagated with the use of the velocity Verlet integrator with a time step of 0.5 fs. On the other hand, the time evolution of the electronic wave function (which is to say, the wave function expansion coefficients) was integrated with the use of the fourth-order Runge–Kutta method with a time step of 0.0004 fs, using quantities interpolated linearly between successive classical time steps.

The electronic state of the system was monitored by following the classical populations of the S_1_ and S_2_ states. As per the usual convention, the classical population of the *j*th adiabatic state from among those included in the FSSH algorithm is defined as the fraction of trajectories that is currently evolving in that state:
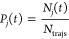
1

Moreover, the twisting of the dimethylamino group of the DMABN molecule was followed by introducing a parameter τ, defined as the average of the absolute values of the dihedral angles formed by atoms C8–N1–C4–C3 and C9–N1–C4–C5:
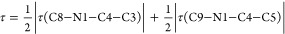
2

### 2.5 Calculation of the TA Spectrum

The working equation for the calculation of a TA spectrum on the basis of molecular dynamics trajectories was given previously by Cerullo and co-workers.^43^ Namely, the signal intensity *I*(*E*, *t*) at photon energy *E* and at time *t* is given by
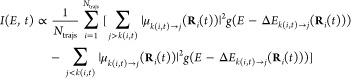
3Here, the index *i* runs over the simulated trajectories, which are *N*_trajs_ in number. The index *k*(*i*, *t*) denotes the current state in the *i*th trajectory at time *t*, and **R**_*i*_(*t*) is the nuclear geometry of the system. The first inner sum is the contribution from ESA and involves states higher than *k*(*i*, *t*). In the present study, this sum was truncated at the S_8_ state. The second inner sum is the contribution from SE and involves states lower than *k*(*i*, *t*), down to and including the ground state (S_0_). μ_*k*(*i*,*t*)→*j*_(**R**_*i*_(*t*)) is the TDM for the transition from state *k*(*i*, *t*) to state *j* at geometry **R**_*i*_(*t*), and Δ*E*_*k*(*i*,*t*)→*j*_ is the energy of that transition. The latter quantity is always taken to be positive, for both upward and downward transitions. Lastly, *g*(*E* – Δ*E*_*k*(*i*,*t*)→*j*_(**R**_*i*_(*t*)) is a line shape function centered at Δ*E*_*k*(*i*,*t*)→*j*_. As a simplifying approximation, the same line shape function was applied for both the ESA and the SE transitions, and it was arbitrarily set to be a Gaussian function. Thus, in the present case, *g*(*E* – Δ*E*_*k*(*i*,*t*)→*j*_(**R**_*i*_(*t*)) is effectively a phenomenological line shape function. In order to obtain a smooth spectrum and also to compensate for the relatively low number of simulated trajectories, its standard deviation was set to the relatively high value of 0.2 eV. Moreover, at the postprocessing stage, the raw simulated spectrum obtained from eq 3 was subjected to a Gaussian blur in the time domain with a standard deviation of σ = 25 fs. Values of *I*(*E*, *t*) were calculated at intervals of 12.5 fs. For the sake of simplicity, the dependence of the TA spectrum on the angle between the probe and pump pulses is neglected, as is the bleaching of the ground state.

On the subject of the choice of the line shape function, it should be noted that several methods are available for the explicit calculation of spectral line shape functions.^75−77^ Our decision to instead use the same phenomenological Gaussian-type line shape function for all transitions is mainly motivated by pragmaticism. Some justification for this approach is provided by the fact that, as we shall see in Sections 3.1 and 3.3, the signatures of the S_1_-LE and S_1_-TICT structures in the TA spectrum are markedly different from one another. Owing to the dissimilarity of their spectral signatures, the approximations used in the calculation of the TA spectrum will not obscure the conversion of the S_1_-LE structure into the S_1_-TICT structure.

Because the TA spectrum is being calculated with a different method (SOS-CC2) than was used in the NAMD simulations (SOS-ADC(2)), an ambiguity arises regarding which state should be considered as the current state *k*(*i*, *t*) for the purpose of calculating the TA spectrum. As an illustration, let us assume that at a certain point in time, the current state in one of the simulated trajectories, calculated with the SOS-ADC(2) method, is the S_2_ state. Which SOS-CC2 excited state should then be taken to be the current state when calculating the TA spectrum: the S_2_ state, or perhaps another state? At some molecular geometries, it may happen that there is no simple one-on-one correspondence, in terms of electronic structure, between SOS-ADC(2) and SOS-CC2 excited states. In order to resolve this ambiguity, we adopted the principle that the SOS-CC2 current state should be whichever state is closest in terms of diabatic character to the SOS-ADC(2) current state. With this in mind, we implemented an automatic state-matching procedure for the assignment of the current state *k*(*i*, *t*). Namely, in order to assign the SOS-CC2 state, we calculated values of the cosine similarity between the singles excitation vectors of the SOS-ADC(2) and SOS-CC2 excited states. The details of this procedure are given in the Supporting Information of ref (7). The SOS-CC2 excited state with the highest cosine similarity to the SOS-ADC(2) current state was then selected as the current state *k*(*i*, *t*) for the purpose of calculating the TA spectrum.

Another complication is that excited-state-to-excited-state TDMs calculated with coupled cluster response methods such as CC2 diverge whenever the energy difference between the initial and final excited states coincides approximately with the energy difference between an excited state and the ground state.^78^ To rephrase this in mathematical notation, μ_*u*→*v*_ diverges whenever Δ*E*_*u*→*v*_ ≈ *ΔE*_0→*w*_, where *u*, *v*, and *w* are any three singlet excited states. (This does also pertain to spin-component scaled variants of CC2, including SOS-CC2.) These divergencies are an artifact of response theory and, if left unchecked, they will overshadow the actual TA spectrum.

However, one factor which works in our favor is that, in the NAMD simulations, the energy gap betwen the S_1_ and S_0_ states (Δ*E*_0→1_) falls predominantly in the range of 2.0–4.5 eV, and it never decreases below 1.5 eV. This is consistent with the fact that the lowest S_1_/S_0_ conical intersection (CI) of DMABN lies relatively high in energy.^79^ Owing to this fortuitous characteristic of the system, the low-energy region of the TA spectrum, up to around 2.0 eV, will be little affected by the unphysical divergencies. Still, they occur frequently in the high-energy region of the spectrum, which is also of interest to us. For this reason, when calculating the TA spectrum, we decided to simply discard all those transitions which are at risk of being affected by such an unphysical divergency. More specifically, any excited-state-to-excited-state transition *u* → *v* was omitted from the TA spectrum if the following condition was met:
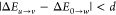
4where *d* is an ad hoc threshold. Naturally, its value should be chosen to be as low as possible, so as to avoid unnecessarily discarding data. By performing some numerical experiments with different values of *d*, we found that the overall appearance of the TA spectrum is only weakly sensitive to the choice of *d* over the range 0.02–0.10 eV. Below that range, the spectrum becomes dominated by the unphysical divergencies; above that range, too much intensity is lost in the high-energy region of the spectrum. (The relationship between the value of *d* and the appearance of the spectrum is further discussed in Section S3 of the Supporting Information.) Accordingly, we decided to set *d* to 0.05 eV. In this regard, we emphasize that the fact that some data had to be discarded means that the intensity scale of the simulated spectrum should be understood to be semiquantitative only.

In closing this section, we note that our methodology for the calculation of the TA spectrum is certainly a pragmatic one, and it relies on there being a relatively large energy gap between the S_1_ and S_0_ states at all times during the NAMD simulations. We expect that it cannot be directly extended to molecules which possess readily accessible S_1_/S_0_ CIs, as the proportion of data points to be discarded would then become too large. This is in addition to the fact that methods such as ADC(2) and CC2 do not provide a formally correct description of crossings between excited states and the ground state.^80^

## 3 Results and Discussion

### 3.1 Benchmark Calculations

Our first order of business was to assess the accuracy of the SOS-CC2 method for the purpose of calculating the TA spectrum of DMABN. To this end, we calculated the vertical absorption and emission spectra of the excited-state structures of the isolated DMABN molecule and compared the results to the benchmark provided by the XMS-CASPT2 method. These calculations were performed at the S_1_-LE and S_1_-TICT excited-state equilibrium geometries optimized at the SOS-ADC(2)/cc-pVDZ level of theory, which were reported previously in ref (7).

The results of the benchmark calculations are summarized in Table 1. All transition energies and oscillator strengths, for both upward and downward transitions, are taken to be positive. We focus first on the S_1_-LE structure, whose transitions are characterized in the upper part of Table 1. The S_1_ → S_0_ transition is responsible for the SE signal of that structure. According to the XMS-CASPT2 calculation, the energy of this transition is 3.822 eV. This coincides closely with the value obtained in the SOS-CC2 calculation, which is 3.958 eV. The SOS-CC2 and XMS-CASPT2 calculations are likewise in good agreement for the energies and intensities of ESA transitions from the S_1_ state into excited states up to, and including, the S_4_ state. Importantly, both methods agree in predicting that the S_1_ → S_3_ transition is the only one from among the lowest four ESA transitions of the S_1_-LE structure to exhibit an appreciably large oscillator strength, although the SOS-CC2 calculation does overestimate the oscillator strength by a factor of roughly 2 relative to the XMS-CASPT2 calculation. This level of accuracy is still acceptable for our purposes because, for reasons explained previously in Section 2.5, the intensity scale of the simulated spectrum is essentially semiquantitative.

**Table 1:** Vertical Transitions of the Excited-State Structures of DMABN, Calculated at the XMS-CASPT2/cc-pVDZ and at the SOS-CC2/cc-pVDZ Levels of Theory: Transition Energies (Δ*E*) and Associated Oscillator Strengths (*f*)^a^

		level of theory			
		XMS-CASPT2/cc-pVDZ		SOS-CC2/cc-pVDZ	
structure	transition	Δ*E*, eV	*f*	Δ*E*, eV	*f*
S_1_-LE	S_1_ → S_0_	3.822	0.020	3.958	0.027
	S_1_ → S_2_	0.703	3 × 10^–5^	0.814	1 × 10^–4^
	S_1_ → S_3_	1.950	0.046	2.113	0.096
	S_1_ → S_4_	2.667	4 × 10^–4^	2.675	0.001
S_1_-TICT	S_1_ → S_0_	2.517	0.112	3.007	0.130
	S_1_ → S_2_	1.138	0.006	1.016	0.007
	S_1_ → S_3_	2.147	0.003	2.136	0.003
	S_1_ → S_4_	2.685	0.002	2.551	0.046

a The calculations were performed at excited-state equilibrium geometries optimized at the SOS-ADC(2)/cc-pVDZ level of theory.

The transitions of the S_1_-TICT structure, in turn, are listed in the lower part of Table 1. For the S_1_ → S_0_ transition, the SOS-CC2 calculation gives an energy of 3.007 eV, while the value from the XMS-CASPT2 calculation is substantially lower, at 2.517 eV. We have no explanation for this large disparity. Interestingly, however, a standard CC2 calculation (without a rescaling of the same- and opposite-spin contributions to the correlation energy) predicts a transition energy of 2.690 eV, considerably lower than the SOS variant and in better agreement with the XMS-CASPT2 benchmark. In this case, it seems that the imposition of the SOS procedure does not achieve a uniform improvement of the accuracy of ADC(2) and CC2 for the ground- and excited-state PESs of DMABN. Rather, there is an improvement for some properties, such as the above-mentioned energy difference between the S_1_-LE and S_1_-TICT structures, but possibly at the cost of degrading accuracy elsewhere.

Another point that requires discussion is the oscillator strength of the S_1_ → S_0_ transition of the S_1_-TICT structure. The XMS-CASPT2 and the SOS-CC2 methods agree in predicting an oscillator strength of around 0.1 for this transition. Despite the close agreement between the two methods, there is reason to believe that the calculated values are overestimated with respect to experiment. This is because of the underlying molecular geometry. As noted above, the benchmark calculations were performed at excited-state equilibrium geometries optimized with the use of the SOS-ADC(2) method. An idiosyncrasy of this method is that, for the S_1_-TICT structure, it predicts an equilibrium geometry where the dimethylamino group adopts a skewed, as opposed to near-perpendicular, orientation with respect to the six-membered ring.^7^ This is unlike some other electronic structure methods, such as CASSCF and the standard variants of ADC(2) and CC2, which predict a near-perpendicular orientation at the equilibrium geometry.^79,81,82^ Because SOS-ADC(2) is a parametrized method, it could be that the skewed S_1_-TICT equilibrium geometry is an artifact of that method and that the true equilibrium geometry is closer to perpendicular. One consequence of the skewed orientation of the dimethylamino group is that the lone pair orbital of its nitrogen atom overlaps fairly well with the out-of-plane p-type orbitals of the ring carbon atoms. This, in turn, imparts a relatively large oscillator strength to the S_1_ → S_0_ transition at the S_1_-TICT structure, considerably larger than at a near-perpendicular orientation, where the overlap is near-zero. (The S_1_ state at the S_1_-TICT structure involves charge transfer from the lone pair orbital dimethylamino group nitrogen atom onto the six-membered ring.) This also has implications for the simulation of the TA spectrum, where the intensity of the SE signal of the S_1_-TICT structure will most likely be overestimated. In other respects, the S_1_-TICT equilibrium geometry predicted by the SOS-ADC(2) method is similar to those obtained with CASSCF and the standard variants of ADC(2) and CC2.

As for excitations from the S_1_ state of the S_1_-TICT structure to the higher excited states, the SOS-CC2 and XMS-CASPT2 methods agree in predicting that both the S_1_ → S_2_ and the S_1_ → S_3_ transitions exhibit low oscillator strengths. As such, these transitions will not make a significant contribution to the TA spectrum. On the other hand, a discrepancy arises regarding the S_1_ → S_4_ transition. According to the XMS-CASPT2 calculation, this transition has a low oscillator strength of 0.002, whereas the SOS-CC2 calculation predicts a fairly large oscillator strength of 0.046. For this reason, we carried out some exploratory XMS-CASPT2 calculations with the inclusion of additional excited states (up to, and including, the S_7_ state), but we did not detect any intense ESA transition at a comparable energy. We therefore conjecture that the high oscillator strength of the S_1_ → S_4_ transition may be an artifact of the SOS-CC2 method. A possible reason for this failure of SOS-CC2 is that the S_1_-TICT structure has a relatively low-energy doubly excited state. This problem is discussed in more detail in Section S4 of the Supporting Information, which also reports the results of extended ADC(2) (ADC(2)-x) calculations for the excited-state structures of DMABN.

In summary, the agreement of the SOS-CC2 method with the XMS-CASPT2 benchmark varies depending on the specific transition in question. Given the somewhat erratic performance of SOS-CC2, it would have been preferable to use instead the XMS-CASPT2 method in the calculation of the TA spectrum. Unfortunately, however, this latter method is too expensive, in terms of CPU time, to be used for that purpose. The best way forward seems to be to use the comparison between the SOS-CC2 and XMS-CASPT2 methods as a guide as to which features of the TA spectrum calculated with the SOS-CC2 method can be deemed realistic and which are presumably artifacts.

**Figure 5:**
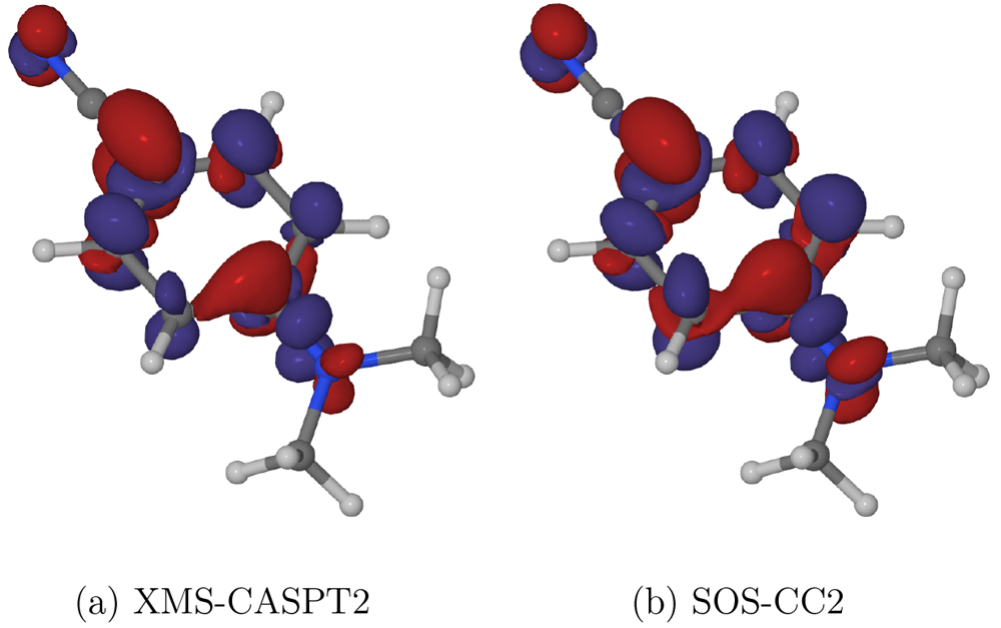
EDDM for the S_1_ → S_3_ transition of the S_1_-LE structure, calculated with the use of the (a) XMS-CASPT2 and (b) SOS-CC2 methods. The EDDM is plotted in form of isosurfaces with isovalues of ±0.0025 *e*/*a*_0_^3^. The red and blue isosurfaces delimit regions in which the electron density is, respectively, increased and decreased upon S_1_ → S_3_ vertical excitation.

One final issue that must be addressed is the character of the S_1_ → S_3_ transition of the S_1_-LE structure. To that end, Figure 5 shows EDDMs for that transition, calculated with the XMS-CASPT2 (panel (a)) and with the SOS-CC2 methods (panel (b)). Both of these methods agree in predicting that the S_1_ → S_3_ transition features a shift of electron density from atoms C2, C3, C5, C6, and the midpoints of the C4–N1 bond onto atoms C1, C4, and N2 (see Figure 1 for atom numbering). In ref (24) this transition was described as involving charge transfer from the dimethylamino group nitrogen (N1) onto the benzonitrile moiety. However, according to the present XMS-CASPT2 and SOS-CC2 calculations, the S_1_ → S_3_ transition does not have a clear-cut charge transfer character. This may be the reason that the simulations reported in ref (24) incorrectly predicted a red shift in the S_1_ → S_3_ transition on going from the gas phase to water. In other respects, our results are largely compatible with those given in ref (24)

### 3.2 Relaxation Dynamics of DMABN in Acetonitrile

We now move on to examine the results of the NAMD simulations which served as the basis for the calculation of the TA spectrum. All 45 simulated trajectories were completed successfully. We did not experience any instances of either the reference RHF calculation or the subsequent SOS-ADC(2) calculation failing to converge. None of the simulated trajectories approached a crossing between the S_1_ and S_0_ states; more specifically, in all simulated trajectories, the S_1_–S_0_ energy gap remained above 1.5 eV at all times.

**Figure 6:**
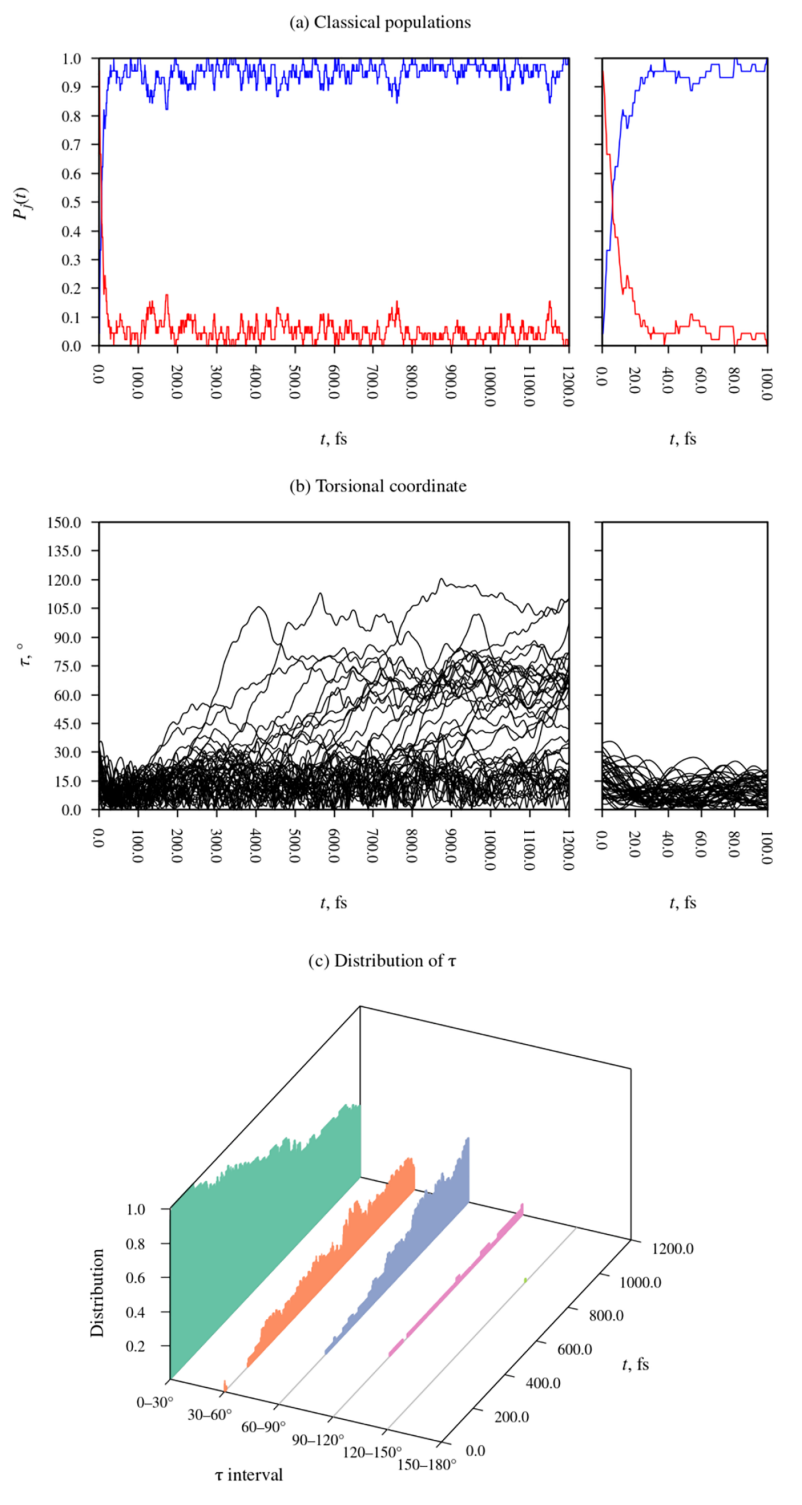
Time evolution of the electronic wave function and molecular geometry during the excited-state relaxation dynamics of DMABN in the 500-molecule acetonitrile nanodroplet. (a) Classical populations of the S_1_ and S_2_ states. Here, and in panel (b), the inset on the right-hand side shows the initial 100 fs long period of the simulations. (b) Values of the parameter τ, which describes the torsional motion of the dimethylamino group, among the ensemble of simulated trajectories. (c) Distribution of the parameter τ, plotted in the form of a histogram.

Figure 6a shows the classical populations of the S_1_ and S_2_ states of DMABN among the ensemble of simulated trajectories. The inset to the right is an enlarged view of the initial 100 fs long period after photoexcitation. The stepped appearance of the plots is a consequence of the fact that when trajectories hop from one state to another, the classical populations inevitably change discontinuously. Figure 6b is a plot of the values of the torsional coordinate τ among the ensemble of simulated trajectories. Figure 6c, in turn, presents the distribution of τ in the form of a histogram. The leftmost, green, bin represents those trajectories in which the value of τ at a given time is in the range from 0° to 30°, the second, orange, bin corresponds to trajectories in which τ is in the range from 30° to 60°, and so on.

At the time of photoexcitation (*t* = 0), the geometries of the DMABN molecule in the ensemble of simulated trajectories were clustered around the ground-state equilibrium geometry (S_0_-GS). 43 of the 45 trajectories comprising the ensemble were initially occupying the S_2_ state, and the remaining two began in the S_1_ state.

Over the 30 fs long period immediately following photoexcitation, the majority of those trajectories which started out in the S_2_ state hopped to the S_1_ state. Afterward, and for the remainder of the simulation period of 1.2 ps, the simulated trajectories predominantly evolved in the S_1_ state. The reason the S_2_ state was not fully depopulated is that individual trajectories occasionally hopped upward from the S_1_ state to the S_2_ state before returning to the S_1_ state a few femtoseconds later. Due to these brief excursions into the S_2_ state, the classical population of that state continuously fluctuated at a level of roughly 0.05.

Following the early S_2_ → S_1_ internal conversion process, the simulation trajectories became trapped in the potential energy basin of the near-planar S_1_-LE structure. From around *t* = 200 fs, in some of the simulated trajectories the DMABN molecule began undergoing a rotation of the dimethylamino group. In Figure 6c, the occurrence of intramolecular rotation can be seen as a gradual decrease in the population of the 0–30° bin, and an increase in the population of the 30–60° bin, and later also of the 60–90° bin. The buildup of the population of the 60–90° bin corresponds to the formation of the S_1_-TICT structure. The simulation time of 1.2 ps was, however, too short for the S_1_-LE and the S_1_-TICT structures to reach equilibrium: the populations of the 0–30° and the 60–90° bins continue changing up until *t* = 1200 fs, with no sign of leveling off.

### 3.3 TA Spectrum

**Figure 7:**
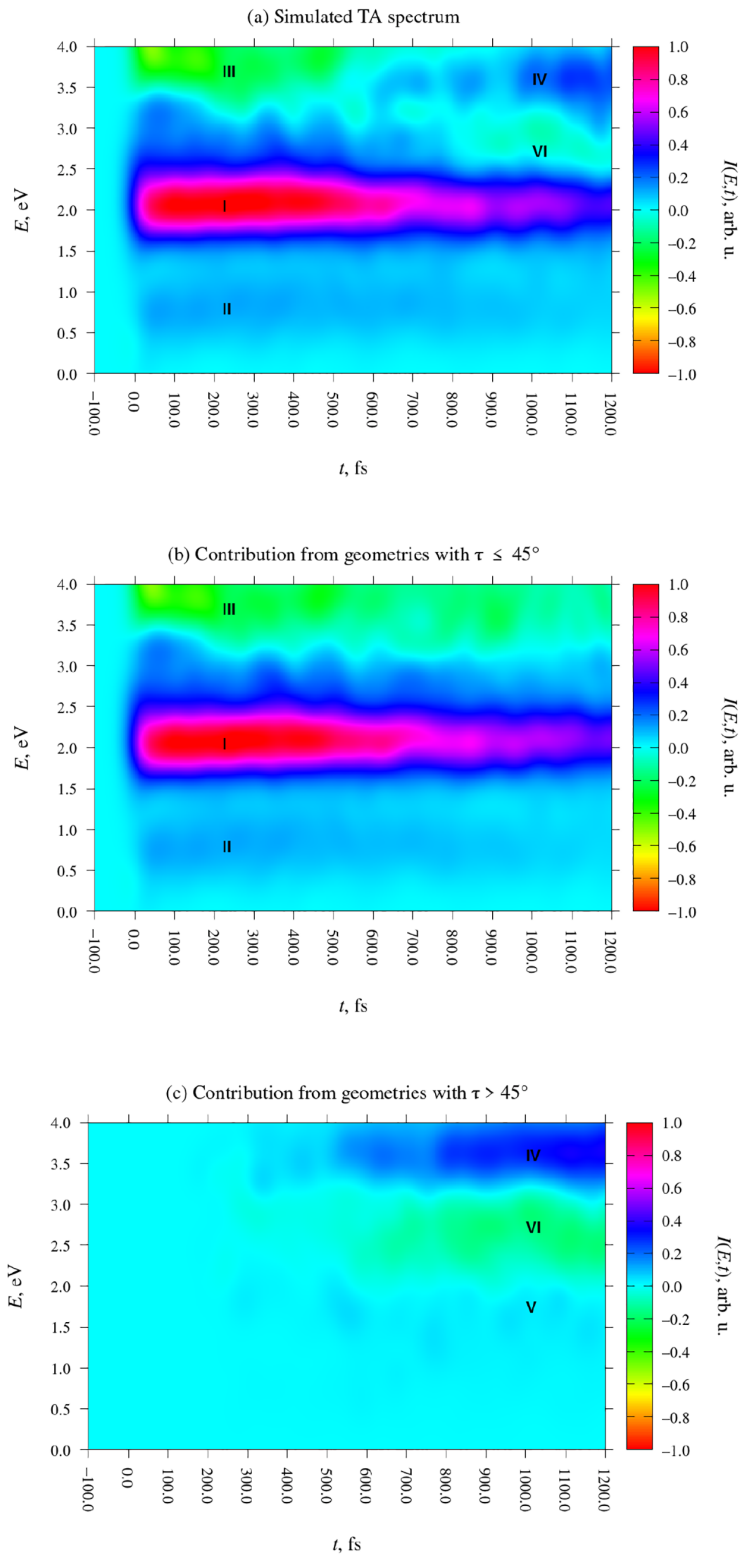
Analysis of the TA spectrum of DMABN in the 500-molecule acetonitrile nanodroplet. Signal intensity, in arbitrary units, is indicated with the use of color. Due to the omission of transitions into doubly excited states and the discarding of some data points, the simulated spectrum is expected to be more accurate in the low-energy range, up to around 2.0 eV, than in the high-energy range. (a) Total signal intensity. The plot is normalized such that the signal maximum corresponds to an intesity of 1. (b) Contribution to signal intensity from geometries with τ ≤ 45°. (c) Contribution to signal intensity from geometries with τ > 45°.

We are now prepared to discuss the main result of our simulations—the TA spectrum of DMABN in acetonitrile. The simulated spectrum is presented in Figure 7a. Its somewhat choppy appearance is an artifact of finite sample size; it was calculated on the basis of a relatively low number (45) of simulated trajectories. In turn, panels (b) and (c) show the decomposition of the spectrum into contributions from molecules with a torsional coordinate τ ≤ 45° and with τ > 45°. The salient features of the spectrum are labeled with Roman numerals.

The most prominent feature of the simulated spectrum is the ESA signal at a photon energy of roughly 2.0 eV, which is labeled **I** in Figure 7. It appears immediately after the initial photoexcitation, and it starts out very intense. Its intensity then gradually decreases over the 1.2 ps long period covered by the simulations. We identify this signal with the ESA signal seen at around 1.8 eV (or 700 nm) in the experimentally observed TA spectrum of DMABN in acetonitrile and other polar solvents.

The origin of this signal can be determined by referring to the breakdown of the spectrum into contributions from molecules with τ ≤ 45° and with τ > 45° (Figure 7b and c, respectively). Indeed, the signal in question is clearly present among the τ ≤ 45° structures but completely absent among the τ > 45° structures. This indicates that it originates from the near-planar S_1_-LE structure. More specifically, cross-referencing with the data in Table 1 shows that this signal arises from the S_1_ → S_3_ transition at the S_1_-LE structure. The gradual decrease of its intensity over time is consistent with the fact that the S_1_-LE structure converts into the S_1_-TICT structure.

The finding that it is the S_1_-LE structure that gives rise to the 1.8 eV (700 nm) ESA signal has implications for the relaxation mechanism of photoexcited DMABN. In several studies, this signal was assigned to the S_1_-RICT structure, a second ICT structure which was held to be nonfluorescent. However, the present simulations not only show that the S_1_-RICT structure does not play a role in the relaxation process but also reveal the true origin of the 1.8 eV (700 nm) ESA signal.

Interestingly, we also see a faint ESA signal in the energy range of roughly 0.5–1.0 eV, which is below the energy range in which the TA spectrum of DMABN has been measured experimentally. InFigure 7, this signal is marked **II**. It arises from the S_1_ → S_2_ transition of the S_1_-LE structure, which is very weak. Similarly to the more intense signal at around 2.0 eV, this feature also decays with time.

The SE signal of the S_1_-LE structure appears in the energy range of roughly 3.5–4.2 eV. It is labeled **III** in Figure 7. In the experimentally observed spectrum, this signal peaks at around 3.4 eV (360 nm),^13^ slightly lower than in our simulations.

The signals due to the S_1_-TICT structure appear later in the simulations. They are difficult to make out in Figure 7a, in part because they overlap with the signals of the S_1_-LE structure, but are readily apparent in Figure 7c, which shows the contribution from structures with τ > 45°. Label **IV** marks a moderately intense ESA signal of the S_1_-TICT structure in the energy range of roughly 3.2–4.0 eV. Experimentally, the ICT structure of DMABN exhibits a sharp ESA signal at 3.9 eV (315 nm),^13^ which is a possible match for the calculated signal. However, the calculated ESA signal of the S_1_-TICT structure must be viewed cautiously because (as discussed in Section S4 of the Supporting Information) the S_1_-TICT structure has a low-lying doubly excited state, which the SOS-CC2 calculation is unable to describe. It is possible that the reasonably good agreement between the observed and calculated TA spectrum in the high-energy range is a coincidence more than anything else.

A weaker ESA signal, which is labeled **V** in Figure 7c, is found at around 1.7 eV. This arises from the S_1_ → S_2_ transition of the S_1_-TICT structure. There is no clear-cut match for this signal in the experimentally observed TA spectrum. According to the analysis by Druzhinin et al.,^13^ the ESA spectrum of the ICT structure of DMABN is almost featureless in the energy range 1.2–2.5 eV (1040–500 nm). The lack of a match is presumably because signal **V** is very weak and was not resolved as a separate feature in the experimentally observed spectrum.

Lastly, the SE signal of the S_1_-TICT structure is labeled **VI**. It appears in the energy range of roughly 2.2–3.2 eV. In the experimentally observed spectrum, this signal is seen in the energy range of some 2.1–2.9 eV (600–430 nm). As discussed previously in Section 3.3, the intensity of the SE signal of the S_1_-TICT structure is most likely overestimated because of the SOS-ADC(2) method having an artificial bias in favor of skewed, rather than fully twisted, molecular geometries.

**Figure 8:**
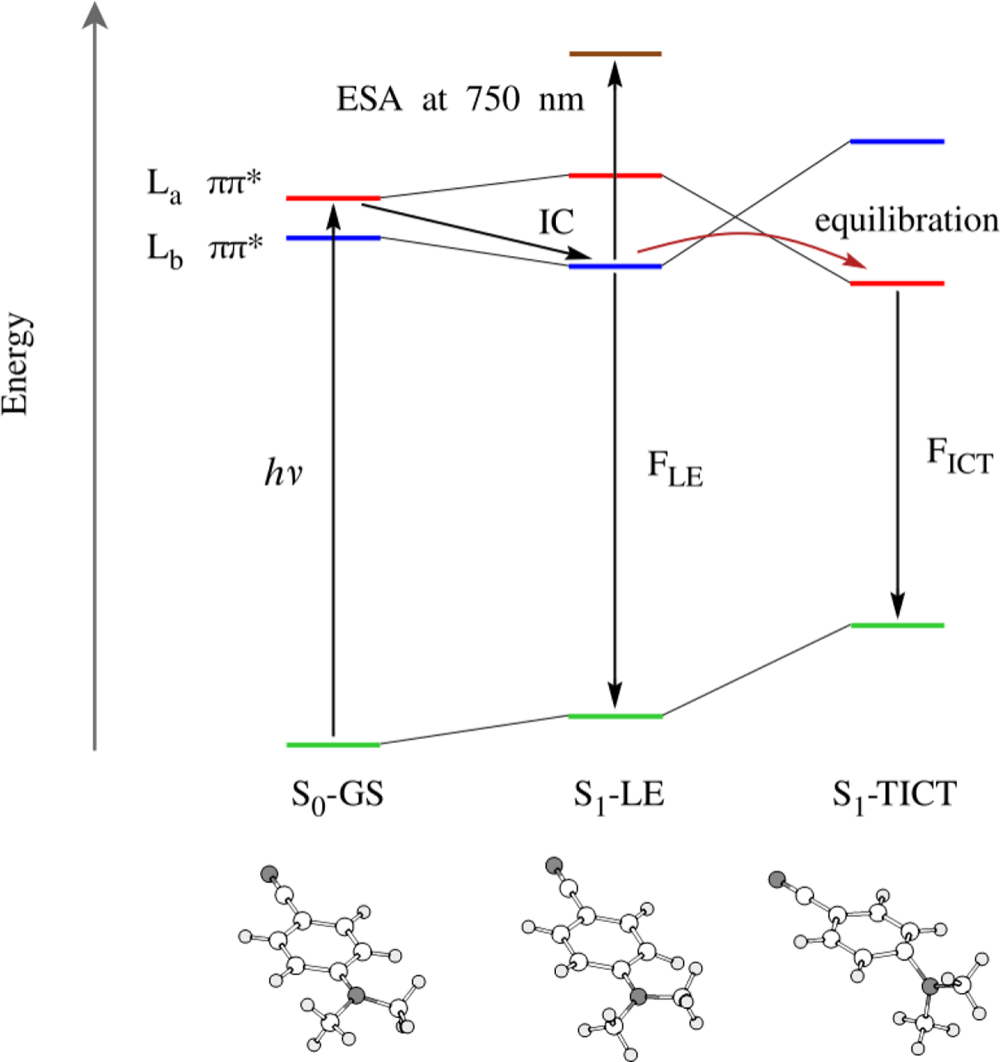
Mechanism of the dual fluorescence of DMABN in acetonitrile solution as predicted by the present simulations. IC denotes the S_2_ → S_1_ internal conversion process. F_LE_ and F_ICT_ are respectively the normal and the anomalous fluorescence processes. The ESA band at 750 nm is found to originate from the S_1_-LE structure.

In Figure 8, information from the analysis of the TA spectrum is incorporated into the overall mechanism of the dual fluorescence of DMABN. The ESA signal at around 750 nm originates from the S_1_ → S_3_ transition of the S_1_-LE structure. It appears immediately after the S_2_ → S_1_ internal conversion process, which is to say, some 20–40 fs after the initial photoexcitation. Its decay is caused by the conversion of the S_1_-LE structure into the S_1_-TICT structure.

## 4 Conclusions

In this study, we set out to simulate and interpret the TA spectrum of DMABN in a polar solvent, a classic model system for the phenomenon of dual fluorescence. To this end, we performed a set of NAMD simulations of the relaxation process of photoexcited DMABN in a 500-molecule acetonitrile nanodroplet. The PESs for this system were constructed with the use of the hybrid QM/MM method, in which the DMABN molecule was treated at the SOS-ADC(2) level of theory. Its dynamics was propagated via the fewest switches surface-hopping algorithm. Afterward, the NAMD trajectories were used as a basis for the calculation of the TA spectrum. At this stage, we resorted to the CC2 method for the calculation of excitation and emission energies and associated TDMs.

The simulated spectrum is in satisfactorily good agreement with the experimentally observed spectrum, allowing a direct comparison between the two. By breaking down the simulated spectrum into contributions from near-planar and twisted molecular geometries, the individual signals can be assigned to specific excited-state structures. Most can also be matched with specific features of the observed spectrum. The only signal which cannot be accounted for is the S_1_ → S_2_ ESA signal of the S_1_-TICT structure, which is, in any case, very weak and might not be identifiable in the observed spectrum. Crucially, our results indicate that the intense ESA signal at around 1.8 eV (700 nm) arises from the S_1_-LE structure and from that structure alone. This finding resolves the long-standing controversy over the assignment of that band.

Last but not least, our study showcases a workflow for the calculation of TA spectra in the ultraviolet–visible range based on NAMD simulations at the ADC(2) level of theory. The caveat is that our approach relies on the CC2 method for the calculation of the TA spectrum itself, and that method can give rise to unphysical divergencies in the calculation of excited-state-to-excited-state TDMs. As a consequence, it is best suited to molecules with long singlet-state lifetimes. Fortunately, however, this category does include many important systems which have been studied with the use of TA spectroscopy, such as fluorescent probes.^83,84^

## Supporting Information Available

Plots of active space orbitals in the CASSCF calculations; additional benchmark calculations; information on the choice of energy threshold for rejecting data points in the calculation of the TA spectrum; results of extended ADC(2) (ADC(2)-x) calculations (PDF)
Animations of simulated trajectories (ZIP)

## Notes

In order to ensure that our results can be reproduced, the NAMD trajectories of DMABN in the 500-molecule acetonitrile nanodroplet are available for download at 10.5281/zenodo.4717368.
