# Supplementary material for: Simulation and Analysis of the Transient Absorption Spectrum of 4-(N,N-Dimethylamino)benzonitrile (DMABN) in Acetonitrile
Source: J Phys Chem A. 2021 Sep 22;125(39):8635–48. doi: 10.1021/acs.jpca.1c06166 (PMC8503879; doi:10.1021/acs.jpca.1c06166)
Supplement: Supplementary file 1 — jp1c06166_si_001.pdf [file jp1c06166_si_001.pdf]

Supporting Information for

Simulation and Analysis of the Transient Absorption Spectrum of 4-(*N*,*N*-Dimethylamino)benzonitrile (DMABN) in Acetonitrile

Michał Andrzej Kochman,[*,†] Bo Durbeej,[‡] and Adam Kubas[†]

[†] Institute of Physical Chemistry, Polish Academy of Sciences, Ul. Marcina Kasprzaka 44/52, 01-224 Warszawa, Poland.
[‡] Division of Theoretical Chemistry, Department of Physics, Chemistry and Biology (IFM), Linköping University, 581 83 Linköping, Sweden

e-mail: mkochman@ichf.edu.pl

Contents

S1 Choice of Active Space in CASSCF Calculations **S2**

S2 Relative Energies of Excited-State Structures **S4**

S3 Choice of Energy Threshold for Rejecting Data Points **S7**

S4 Extended ADC(2) (ADC(2)-x) Calculations **S10**

References **S12**

S1 Choice of Active Space in CASSCF Calculations

In order to ensure that our results can be reproduced, Figure S1 shows plots of the active space orbitals employed in the CASSCF calculations for DMABN.

Figure S1: CASSCF active space natural orbitals of DMABN, plotted in the form of isosurfaces with isovalues of $\pm 0.05\ a_0^{-3/2}$. The orbitals were generated through a SA-5-CASSCF(12,11)/cc-pVDZ calculation at the S_1-LE geometry optimized at the SOS-ADC(2)/cc-pVDZ level of theory.

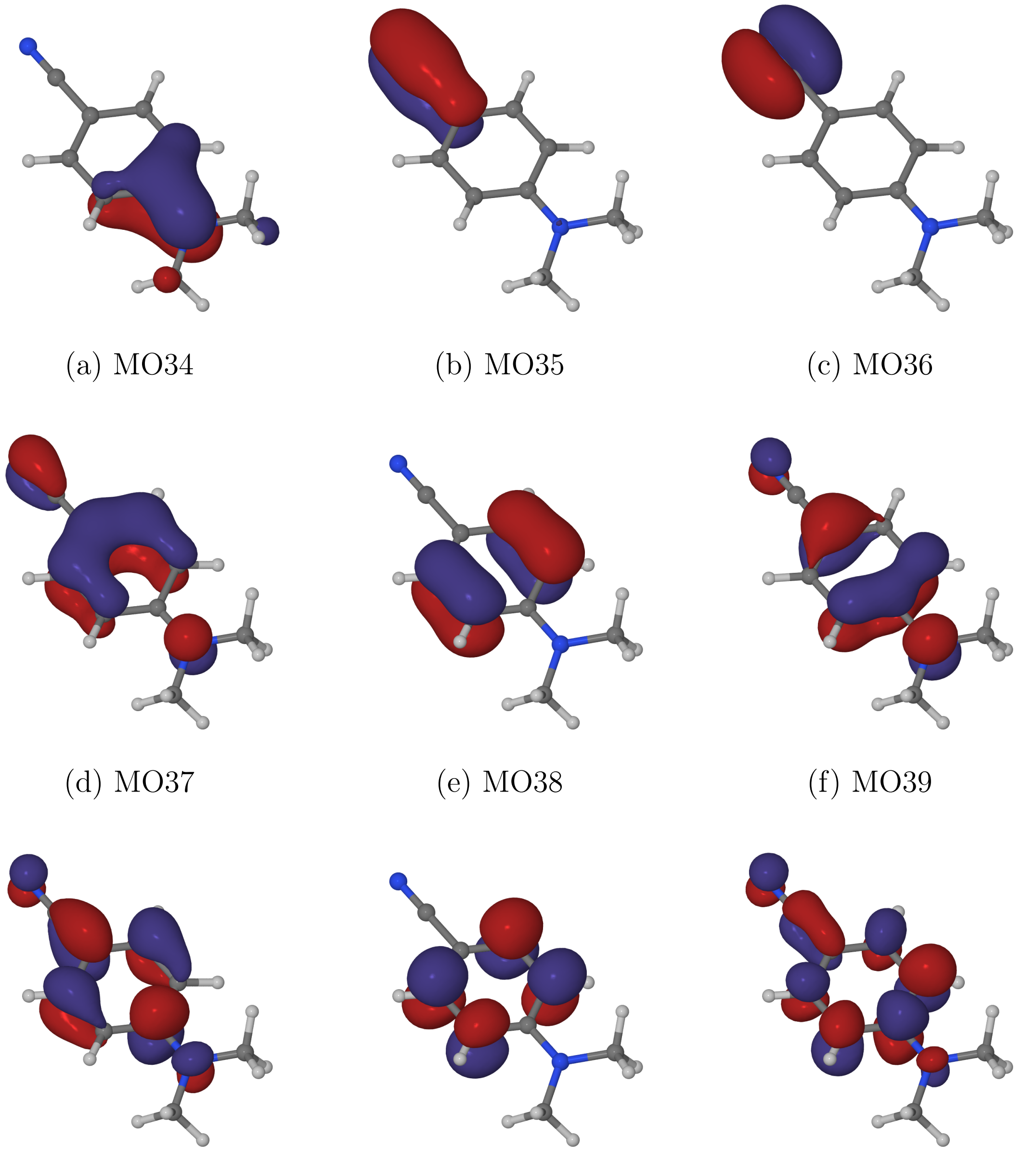

(a) MO34 (b) MO35 (c) MO36

(d) MO37 (e) MO38 (f) MO39

(g) MO40 (h) MO41 (i) MO42

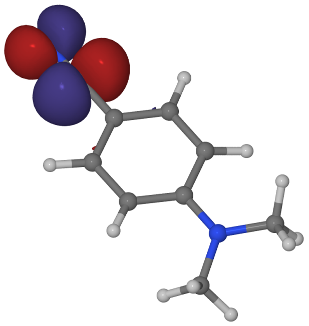

(j) MO43

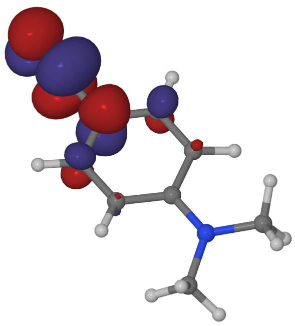

(k) MO44

S2 Relative Energies of Excited-State Structures

In Section 3.1 of our paper, we employed the XMS-CASPT2 method as a benchmark against which to evaluate the performance of the SOS-CC2 method for the calculation of ESA transitions of DMABN. In the present section, we use XMS-CASPT2 to calculate the relative energies of the various excited-state structures of that molecule, with special regard to the question of whether the S_1-RICT structure is involved in its excited-state relaxation mechanism.

For the sake of consistency with the benchmark calculations performed previously in Ref.,[12] the XMS-CASPT2 calculations were performed as single-point calculations of energy at excited-state equilibrium geometries of DMABN as optimized at the SOS-ADC(2)/cc-pVTZ level of theory. The calculation settings were the same as specified in Section 2.2 of our paper. In what follows, we refer to this composite level of theory (XMS-CASPT2 single-point energies at SOS-ADC(2)-optimized geometries) as XMS-CASPT2//SOS-ADC(2).

Several studies[13–22] have investigated the photophysics of DMABN with variants of the time-dependent density functional theory (TDDFT) method. For this reason, it is also of interest to see how the predictions of TDDFT compare with the wavefunction-based methods. To this end, we also calculated the energies of the excited-state structures of DMABN with TDDFT. These calculations were performed with the computational chemistry software package Gaussian 16, Revision A.03.[23] We elected to use the CAM-B3LYP functional,[24] because that functional was used previously to investigate the excited-state potential energy surfaces[19,20] and excited-state relaxation dynamics[21] of DMABN. The 6-311G(d) basis set[25] was employed. This calculation is denoted as TD-CAM-B3LYP//SOS-ADC(2).

The results are summarized in Table S1 on the following page, which also includes the ADC(3)//SOS-ADC(2) energies reported previously in Ref.[12] For ease of reference, the same data is presented visually in Figure S2 on page S6.

The SOS-ADC(2) and ADC(3)//SOS-ADC(2) calculations predict the same energy ordering of the excited-state structures of DMABN, with the S_1-LE structure being the lowest in energy, the S_1-TICT structure being somewhat higher, and the S_1-RICT substantially higher.

The XMS-CASPT2//SOS-ADC(2) calculation places the S_1-TICT slightly below the S_1-LE structure (lower by 0.100 eV). We have no definitive explanation for why, at this level of theory, the energy ordering of the S_1-TICT and the S_1-LE structures is inverted with respect to the SOS-ADC(2) and the ADC(3)//SOS-ADC(2) calculations. One possibility is that the relatively small basis set (cc-pVDZ) employed in the XMS-CASPT2 calculations is artificially stabilizing the S_1-TICT structure relative to the S_1-LE structure. In fact, precisely such an effect was previously reported to occur with the CC2 method.[26] However, it is unclear whether this behavior can be extrapolated from the CC2 method to the XMS-CASPT2 method.

Importantly, we find that the SOS-ADC(2), ADC(3)//SOS-ADC(2), and XMS-CASPT2//SOS-ADC(2) calculations all agree in predicting that the S_1-RICT structure lies considerably higher in energy than either of the S_1-TICT and S_1-LE structures. This rules out the possibility that the S_1-RICT structure plays a role in the relaxation mechanism of photoexcited DMABN.

Table S1: Comparison of relative energies of the excited-state structures of DMABN calculated at different levels of electronic structure theory: SOS-ADC(2), ADC(3)//SOS-ADC(2), XMS-CASPT2//SOS-ADC(2), and TD-CAM-B3LYP//SOS-ADC(2). The energy values are given relative to the energy of the S_0-GS structure at the given level of theory, and include zero-point vibrational energy (ZPVE) corrections calculated at the SOS-ADC(2) level. See text for details of each calculation.

Level of theory	E, eV		
	S_1-LE	S_1-TICT	S_1-RICT
SOS-ADC(2)	4.080	4.296	5.414
ADC(3)//SOS-ADC(2)	4.116	4.423	5.031
XMS-CASPT2//SOS-ADC(2)	3.938	3.838	5.134
TD-CAM-B3LYP//SOS-ADC(2)	4.446	4.405	4.742

Lastly, according to the TD-CAM-B3LYP//SOS-ADC(2) calculation, the S_1-TICT structure lies marginally lower in energy (by 0.041 eV) than the S_1-LE structure. The S_1-RICT structure is predicted to be higher in energy, but still fairly close, to the other two excited-state structures. (The energy separation between the S_1-RICT structure and the other two is only around 0.3 eV.) If the results of the TD-CAM-B3LYP//SOS-ADC(2) calculation are taken at face value, one might expect the S_1-RICT structure to be populated, at least to a small degree, during the relaxation mechanism of photoexcited DMABN. However, the small energy gap between the S_1-RICT structure and the other two excited-state structures is most likely an artifact of the TD-CAM-B3LYP method, as all three wavefunction-based methods considered here indicate that the S_1-RICT structure actually lies substantially higher in energy, and is effectively inaccessible.

Figure S2: Energy level diagram for DMABN obtained with different levels of theory. The origin of the energy scale is set to the energy of the S_0-GS structure at the given level of theory. The energies include ZPVE corrections calculated at the SOS-ADC(2)/cc-pVTZ level.

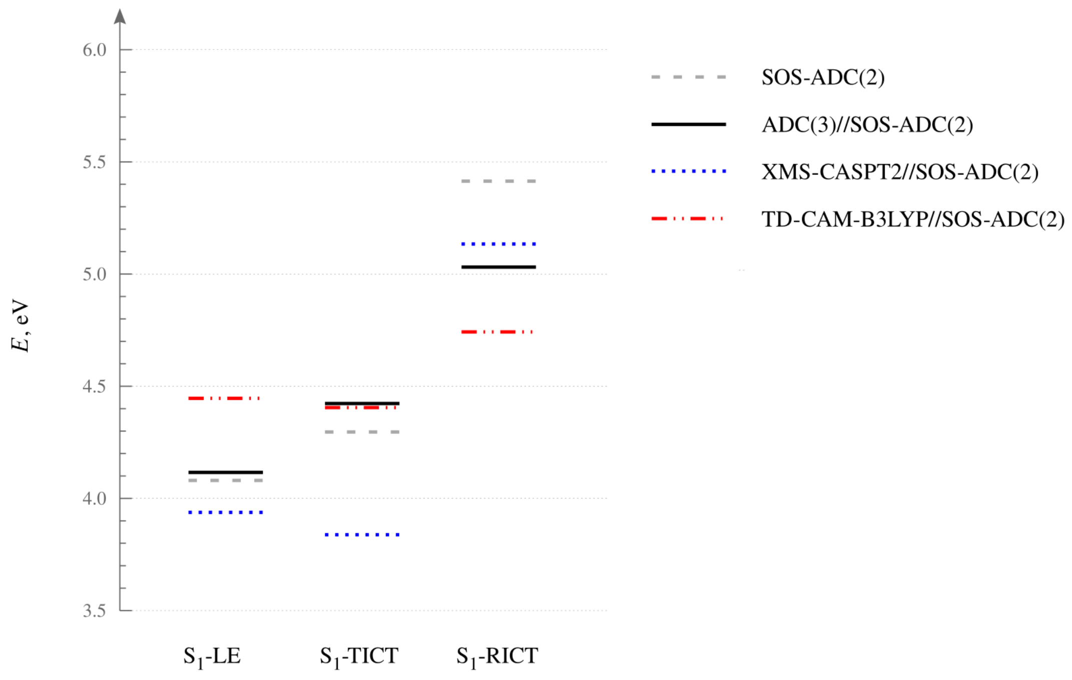

S3 Choice of Energy Threshold for Rejecting Data Points

As mentioned in Section 2.5 of our paper, excited-state-to-excited-state TDMs calculated with the use of the CC2 method diverge whenever the energy difference between the initial and final excited states coincides approximately with the energy difference between an excited state and the ground state. In practice, these divergencies manifest themselves as extremely large TDM magnitudes.

We mitigated this problem by discarding all transitions which were potentially at risk of being affected by an unphysical divergence. More specifically, any excited-state-to-excited-state transition $u \to v$ was omitted from the TA spectrum if the following condition was met:

$$|\Delta E_{u\to v} - \Delta E_{0\to w}| < d \tag{1}$$

Here, u, v, and w are any three singlet excited states at a given nuclear geometry of the system, and d is a predefined energy threshold.

Clearly, the value of d must be chosen to be as low as possible, but still high enough that all of the unphysical divergencies are removed. It can be argued that once the divergencies have been discarded, the overall appearance of the simulated TA spectrum should be fairly insensitive to further increases in the value of d. This is because any further increase in d will only eliminate transitions with realistic, low to moderately large TDMs, and not transitions with artificial and extremely large TDMs. With that in mind, we investigated the relationship between the choice of d and the appearance of the simulated spectrum.

Figure S3 shows simulated TA spectra obtained with different values of d in the range of $0-0.10$ eV. (Zero means that no data was discarded) At $d = 0$, we see several sharp and very intense peaks in the energy range of around $2.0-4.0$ eV. These arise purely from divergencies in the calculated TDMs, and are simulation artifacts. On the other hand, the low-energy range of the spectrum (up to around 2.0 eV) is free from artifacts. This is because the condition $\Delta E_{u\to v} \approx \Delta E_{0\to w}$ is not satisfied in that energy range.

The imposition of an already low, non-zero energy threshold ($d = 0.01$ eV) eliminates most of the offending peaks. Increasing d further, up to 0.10 eV, has the effect of weakening the ESA signal of the S_1-TICT structure, which appears at an energy of roughly 3.5 eV starting from around $t = 500$ fs, but the spectrum is otherwise little affected. We therefore decided to set $d = 0.05$ eV, which should ensure that all of the artificial signals in the energy range of $2.0-4.0$ eV is eliminated, but without discarding too much of the real data.

Figure S3: Plots of simulated TA spectra of DMABN in acetonitrile obtained with different values of d. All plots are normalized to the same intensity scale.

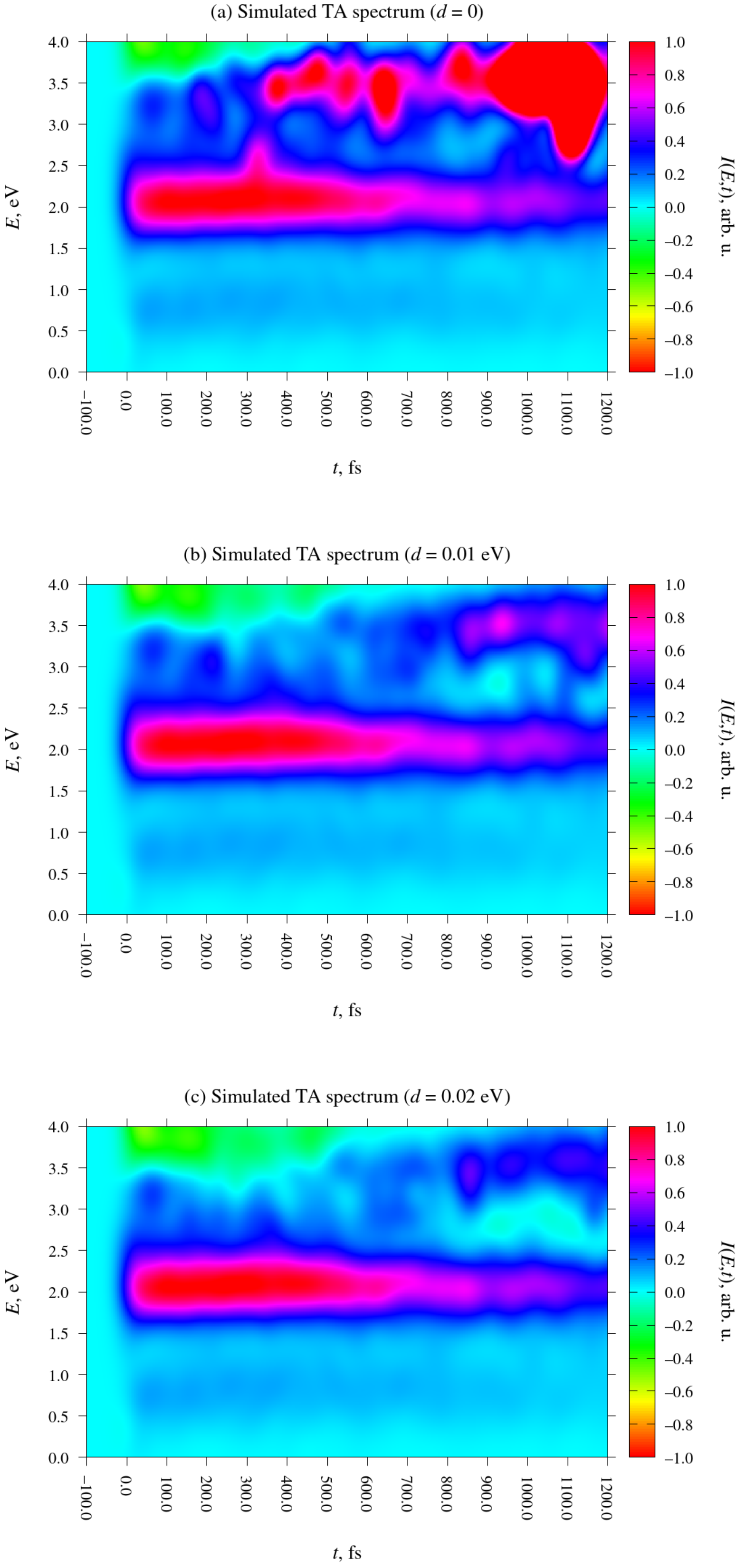

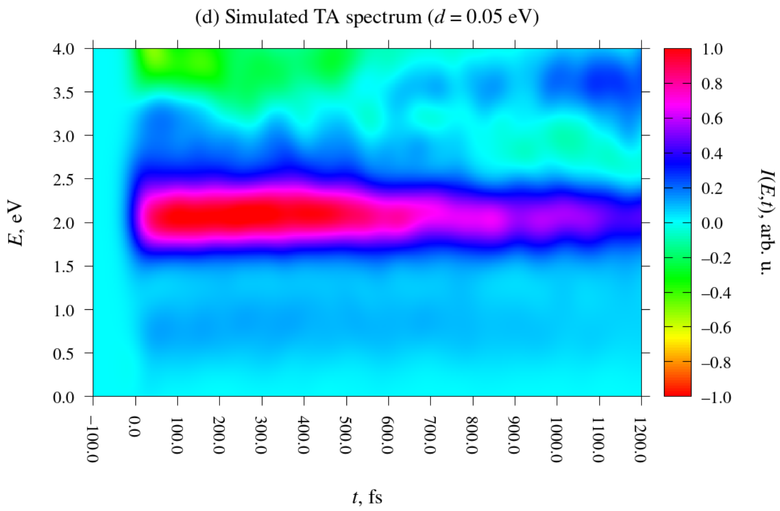

(d) Simulated TA spectrum (d = 0.05 eV)
E, eV
t, fs
I(E,t), arb. u.

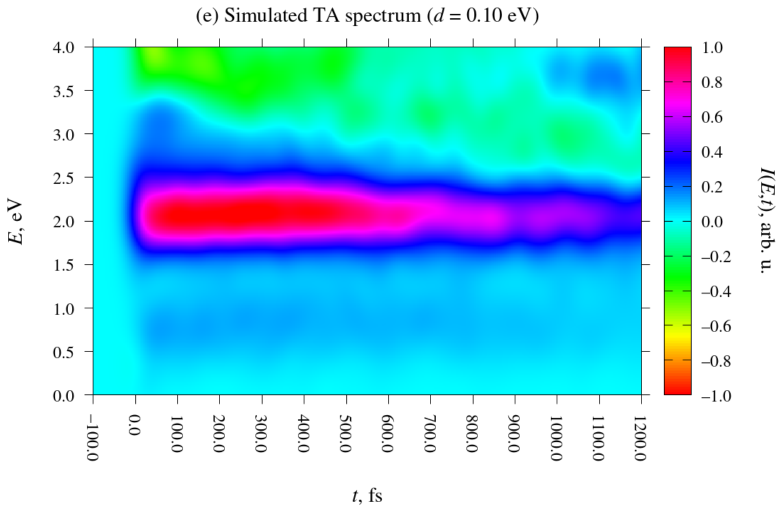

(e) Simulated TA spectrum (d = 0.10 eV)
E, eV
t, fs
I(E,t), arb. u.

S4 Extended ADC(2) (ADC(2)-x) Calculations

Methods such as CC2 and the standard variant of ADC(2) (which in this context is called 'strict' ADC(2), or ADC(2)-s) are only capable of providing a realistic description of those excited states which are dominated by single excitations from the reference determinant. They are unable to describe states with a significant contribution from double or higher excitations. This is part of the reason that we assessed the performance of the SOS-CC2 method for the calculation of ESA and SE transitions of DMABN against the benchmark provided by the XMS-CASPT2 method, which is able to give a correct description of doubly-excited states. Another issue was the quantitative accuracy of the CC2 method − even for transitions into states dominated by single excitations, CC2 can give errors of up to a few tenths of an electronvolt.[1–6]

While XMS-CASPT2 is invaluable in providing accurate excitation energies and oscillator strengths, with that method it is not straightforward to quantify the contribution of single, double, etc. excitations to a given excited state. Clearly, it would be very useful to have that information, as it would help understand the performance of CC2. Accordingly, we have performed another set of benchmark calculations, in which the excited electronic states of DMABN were described with the use of the extended ADC(2)[7,8] method (ADC(2)-x). ADC(2)-x is an *ad hoc* modification of ADC(2)-s designed to enable the description of doubly-excited states. In ADC(2)-x, the two particle-two hole block of the ADC matrix is expanded up to first order of perturbation theory, higher than in ADC(2)-s, where it is expanded to zeroth order (and is diagonal). This measure enables the calculation of doubly-excited states, though at the cost of introducing a certain imbalance between singly- and doubly-excited states, and a systematic underestimation of excitation energies. Because ADC(2)-x consistently underestimates excitation energies, it is not well suited for comparisons with experiment, but it is still useful as a diagnostic tool for the detection of doubly-excited states. Specifically, the singly vs doubly excited character of a given state can be quantified by calculating the square norm of the singles ($|\mathbf{v}_1|^2$) and the doubles ($|\mathbf{v}_2|^2$) parts of its excitation vector.

The ADC(2)-x calculations were performed with the program ADC-connect.[9,10] The reference RHF calculations were done with the program PySCF,[11] which is interfaced to ADC-connect. Spin-component scaling was not imposed in the ADC(2)-x calculations.

The results are summarized in Table S2. For the S_1-LE structure, we find that states S_1 to S_3 are all dominated by single excitations. The fact that all three are essentially singly-excited states is very encouraging, as it confirms that CC2 will be reliable for the $S_1 \rightarrow S_3$ transition of the S_1-LE structure, which, according to our calculations, is responsible for the important ESA band near 1.7 eV. In the case of the S_4 state, the contribution from doubly-excited configurations is somewhat larger than for the lower three states, with $|\mathbf{v}_2|^2 = 0.32$, but it is still relatively small.

For the S_1-TICT structure, in turn, states S_1 to S_3 are again essentially singly excited states, with minor contributions from doubly-excited configurations. The S_4 state, however, has substantial doubly-excited character, with $|\mathbf{v}_2|^2 = 0.55$. The inspection of its excitation vector reveals that its doubly excited character arises in large part from the $(\text{HOMO})^0(\text{LUMO})^2$ configuration. The CC2 method

Table S2: Characterization of the excited electronic states of DMABN at the ADC(2)-x/cc-pVDZ level of theory. $E(\mathrm{S}_n) - E(\mathrm{S}_0)$ is the vertical energy gap between a given excited state, and the singlet ground state. $|\mathbf{v}_1|^2$ and $|\mathbf{v}_2|^2$ are, respectively, the norms of the singles and doubles parts of the excitation vector of the given excited state. The calculations were performed at excited-state equilibrium geometries optimized at the SOS-ADC(2)/cc-pVDZ level of theory.

Structure	State	$E(\mathrm{S}_n) - E(\mathrm{S}_0)$, eV	$\lvert\mathbf{v}_1\rvert^2$	$\lvert\mathbf{v}_2\rvert^2$
S_1-LE	S_1	3.011	0.82	0.18
	S_2	3.838	0.82	0.18
	S_3	4.790	0.76	0.24
	S_4	5.662	0.68	0.32
S_1-TICT	S_1	1.935	0.85	0.15
	S_2	2.990	0.80	0.20
	S_3	3.932	0.80	0.20
	S_4	4.170	0.45	0.55

(including its SOS-CC2 variant) will not be capable of providing a correct description of that state. In fact, in the CC2 calculation, the error is not necessarily restricted to a single excited state, but may potentially affect multiple states. This is because of the orthonormality condition: all excited states are required to be mutually orthonormal, but at least one has an incorrect structure because of the missing contribution from doubly excited configurations. Hence, there may be an indirect (or, 'knock-on') effect on some of the other states.

In summary, the results of the ADC(2)-x calculations indicate that the CC2 method is reliable for at least the lowest three, and possibly also for the fourth, of the singlet excited states of the S_1-LE structure. The situation seems somewhat worse for the S_1-TICT structure, as the ADC(2)-x calculations reveal the presence of a fairly low-energy state with a partial doubly-excited character. For this reason, the contribution of twisted molecular geometries to the calculated TA spectrum must be interpreted cautiously.

References

[1] Hellweg, A.; Grün, S. A.; Hättig, C. Benchmarking the Performance of Spin-Component Scaled CC2 in Ground and Electronically Excited States *Phys. Chem. Chem. Phys.* **2008**, *10*, 4119-4127. DOI: 10.1039/b803727b

[2] Szalay, P. G.; Watson, T.; Perera, A.; Lotrich, V. F.; Bartlett, R. J. Benchmark Studies on the Building Blocks of DNA. 1. Superiority of Coupled Cluster Methods in Describing the Excited States of Nucleobases in the Franck-Condon Region *J. Phys. Chem. A* **2012**, 116, 6702-6710. DOI: 10.1021/jp300977a

[3] Winter, N. O. C.; Graf, N. K.; Leutwyler, S.; Hättig, C. Benchmarks for 0−0 Transitions of Aromatic Organic Molecules: DFT/B3LYP, ADC(2), CC2, SOS-CC2 and SCS-CC2 Compared to High-Resolution Gas-Phase Data. *Phys. Chem. Chem. Phys.* **2013**, *15*, 6623-6630. DOI: 10.1039/C2CP42694C

[4] Tajti, A.; Szalay, P. G. Accuracy of Spin-Component-Scaled CC2 Excitation Energies and Potential Energy Surfaces. *J. Chem. Theory Comput.* **2019**, *15*, 5523-5531. DOI: 10.1021/acs.jctc.9b00676

[5] Tajti, A.; Tulipán, L.; Szalay, P. G. Accuracy of Spin-Component Scaled ADC(2) Excitation Energies and Potential Energy Surfaces. *J. Chem. Theory Comput.* **2020**, *16*, 468-474. DOI: 10.1021/acs.jctc.9b01065

[6] Oruganti, B.; Fang, C.; Durbeej, B. Assessment of a Composite CC2/DFT Procedure for Calculating 0−0 Excitation Energies of Organic Molecules. *Mol. Phys.* **2016**, *114*, 3448-3463. DOI: 10.1080/00268976.2016.1235736

[7] Trofimov, A. B.; Schirmer, J. An Efficient Polarization Propagator Approach to Valence Electron Excitation Spectra. *J. Phys. B: At., Mol. Opt. Phys.* **1995**, *28*, 2299-2324, DOI: 10.1088/0953-4075/28/12/003

[8] Dreuw, A.; Wormit, M. The Algebraic Diagrammatic Construction Scheme for the Polarization Propagator for the Calculation of Excited States. *WIREs Comput. Mol. Sci.* **2015**, *5*, 82-95. DOI: 10.1002/wcms.1206

[9] https://adc-connect.org/v0.13.3/index.html , accessed on March 1, 2021.

[10] Herbst, M. F.; Scheurer, M. adc-connect/adcc v0.13.4 (Version v0.13.4). 2020 Zenodo. DOI: 10.5281/zenodo.3631806.

[11] Sun, Q.; Berkelbach, T. C.; Blunt, N. S.; Booth, G. H.; Guo, S.; Li, Z.; Liu, J.; McClain, J. D.; Sayfutyarova, E. R.; Sharma, S.; Wouters, S.; Chan, G. K.-L. PySCF: the Python-Based Simulations of Chemistry Framework. *WIREs Comput. Mol. Sci.* **2018**, *8*, e1340. DOI: 10.1002/wcms.1340

[12] Kochman, M. A.; Durbeej, B. Simulating the Nonadiabatic Relaxation Dynamics of 4-(*N*,*N*-Dimethylamino)benzonitrile (DMABN) in Polar Solution. *J. Phys. Chem. A* **2020**, *124*, 2193-2206. DOI: 10.1021/acs.jpca.9b10588

[13] Parusel, A. B. J.; Rettig, W.; Sudholt, W. A Comparative Theoretical Study on DMABN: Significance of Excited State Optimized Geometries and Direct Comparison of Methodologies. *J. Phys. Chem. A* **2002**, *106*, 804-815. DOI: 10.1021/jp015513m

[14] Rappoport, D.; Furche, F. Photoinduced Intramolecular Charge Transfer in 4-(Dimethyl)aminobenzonitrile – A Theoretical Perspective. *J. Am. Chem. Soc.* **2004**, *126*, 1277-1284. DOI: 10.1021/ja037806u

[15] Zgierski, M. Z.; Lim, E. C. The Role of $\pi\sigma^*$ State in Intramolecular Electron-Transfer Dynamics of 4-Dimethylaminobenzonitrile and Related Molecules. *J. Chem. Phys.* **2004**, *121*, 2462-2465. DOI: 10.1063/1.1780154

[16] Zgierski, M. Z.; Lim, E. C. Electronic and Vibrational Spectra of the Low-Lying $\pi\sigma^*$ State of 4-dimethylaminobenzonitrile: Comparison of Theoretical Predictions with Experiment. *J. Chem. Phys.* **2005**, *122*, 111103. DOI: 10.1063/1.1889431

[17] Chiba, M.; Tsuneda, T.; Hirao, K. Long-Range Corrected Time-Dependent Density Functional Study on Fluorescence of 4,4′-Dimethylaminobenzonitrile. *J. Chem. Phys.* **2007**, *126*, 034504. DOI: 10.1063/1.2426335

[18] Zhao, G.-J.; Han, K.-L. Time-Dependent Density Functional Theory Study on Hydrogen-Bonded Intramolecular Charge-Ttransfer Excited State of 4-Dimethylamino-benzonitrile in Methanol. J. Comp. Chem. **2008**, *29*, 2010-2017. DOI: 10.1002/jcc.20957

[19] Wiggins, P.; Williams, J. A. G.; Tozer, D. J. Excited State Surfaces in Density Functional Theory: A New Twist on an Old Problem. *J. Chem. Phys.* **2009**, *131*, 091101. DOI: 10.1063/1.3222641

[20] Zhong, C. The Driving Forces for Twisted or Planar Intramolecular Charge Transfer. *Phys. Chem. Chem. Phys.* **2015**, *17*, 9248-9257. DOI: 10.1039/C4CP02381A

[21] Du, L.; Lan, Z. An On-the-Fly Surface-Hopping Program JADE for Nonadiabatic Molecular Dynamics of Polyatomic Systems: Implementation and Applications. *J. Chem. Theory Comput.* **2015**, *11*, 1360-1374. DOI: 10.1021/ct501106d

[22] Curchod, B. F. E.; Sisto, A.; Martínez, T. J. Ab Initio Multiple Spawning Photochemical Dynamics of DMABN Using GPUs. *J. Phys. Chem. A* **2017**, *121*, 265-276. DOI: 10.1021/acs.jpca.6b09962

[23] Gaussian 16, Revision A.03, Frisch, M. J.; Trucks, G. W.; Schlegel, H. B.; Scuseria, G. E.; Robb, M. A.; Cheeseman, J. R.; Scalmani, G.; Barone, V.; Petersson, G. A.; Nakatsuji, H.; Li, X.; Caricato, M.; Marenich, A. V.; Bloino, J.; Janesko, B. G.; Gomperts, R.; Mennucci, B.; Hratchian, H. P.; Ortiz, J. V.; Izmaylov, A. F.; Sonnenberg, J. L.; Williams-Young, D.; Ding, F.; Lipparini, F.; Egidi, F.; Goings, J.; Peng, B.; Petrone, A.; Henderson, T.; Ranasinghe, D.; Zakrzewski, V. G.; Gao, J.; Rega, N.; Zheng, G.; Liang, W.;

Hada, M.; Ehara, M.; Toyota, K.; Fukuda, R.; Hasegawa, J.; Ishida, M.; Nakajima, T.; Honda, Y.; Kitao, O.; Nakai, H.; Vreven, T.; Throssell, K.; Montgomery, J. A., Jr.; Peralta, J. E.; Ogliaro, F.; Bearpark, M. J.; Heyd, J. J.; Brothers, E. N.; Kudin, K. N.; Staroverov, V. N.; Keith, T. A.; Kobayashi, R.; Normand, J.; Raghavachari, K.; Rendell, A. P.; Burant, J. C.; Iyengar, S. S.; Tomasi, J.; Cossi, M.; Millam, J. M.; Klene, M.; Adamo, C.; Cammi, R.; Ochterski, J. W.; Martin, R. L.; Morokuma, K.; Farkas, O.; Foresman, J. B.; Fox, D. J. Gaussian, Inc., Wallingford CT, 2016.

[24] Yanai, T.; Tew, D.; Handy, N. A New Hybrid Exchange-Correlation Functional Using the Coulomb-Attenuating Method (CAM-B3LYP). *Chem. Phys. Lett.* **2004**, *393*, 51-57. DOI: 10.1016/j.cplett.2004.06.011

[25] Krishnan, R.; Binkley, J. S.; Seeger, R.; Pople, J. A. Self-Consistent Molecular Orbital Methods. XX. A Basis Set for Correlated Wave Functions *J. Chem. Phys.* **1980**, *72*, 650-654. DOI: 10.1063/1.438955

[26] Köhn, A.; Hättig, C. On the Nature of the Low-Lying Singlet States of 4-(Dimethyl-amino)benzonitrile *J. Am. Chem. Soc.* **2004**, *126*, 7399-7410. DOI: 10.1021/ja0490572
